# miR-221/222 Targets Adiponectin Receptor 1 to Promote the Epithelial-to-Mesenchymal Transition in Breast Cancer

**DOI:** 10.1371/journal.pone.0066502

**Published:** 2013-06-11

**Authors:** Michael S. Hwang, Nancy Yu, Susanna Y. Stinson, Peng Yue, Robert J. Newman, Bernard B. Allan, David Dornan

**Affiliations:** 1 Department of Molecular Diagnostics and Cancer Cell Biology, Genentech, Inc., South San Francisco, California, United States of America; 2 Department of Bioinformatics and Computational Biology, Genentech, Inc., South San Francisco, California, United States of America; 3 Department of Molecular Biology, Genentech, Inc., South San Francisco, California, United States of America; AMS Biotechnology, United Kingdom

## Abstract

The epithelial-to-mesenchymal transition (EMT) is a highly conserved physiological program involved in development and tissue repair; however, its aberrant activation has been implicated in accelerating the progression of a variety of cancers. In breast cancer, the microRNAs (miRNAs) miR-221 and miR-222 (miR-221/222) are differentially expressed in the clinically more aggressive basal-like subtype compared to luminal subtype of breast cancer and upregulation of miR-221/222 induces the EMT by targeting the 3′ untranslated region (3′UTR) of the GATA family transcriptional repressor TRPS1 (tricho-rhino-phalangeal syndrome type 1). The complete mechanism through which miR-221/222 promotes the EMT, however, is not fully understood. We identified adiponectin receptor 1 (ADIPOR1), a receptor for the adipocytokine adiponectin, as a direct target of miR-221/222. ADIPOR1 is expressed at higher levels in the luminal compared to the basal-like subtype of breast cancer cell lines, which can be reduced by miR-221/222 targeting of its 3’UTR. In addition, miR-221/222 were negatively correlated with ADIPOR1 expression across breast cancer cell lines and tumors. ADIPOR1 depletion by siRNA in MCF10A cells induced the EMT and increased cell invasion. Depletion of ADIPOR1 by siRNA induced activation of the canonical nuclear factor-kappaB (NF-κB) and subsequent phosphorylation of signal transducer and activator of transcription 3 (STAT3) in an interleukin 6 (IL6)-dependent manner. Finally, overexpression of ADIPOR1 in the basal-like cell line, MDA-MB-231, attenuated cell invasion and promoted the mesenchymal-to-epithelial transition (MET). We conclude that ADIPOR1 negatively regulates EMT in breast cancer and provides an additional node by which miR-221/222 induces the EMT. These results suggest that ADIPOR1 may play an important role in breast cancer progression and metastasis, and could potentially offer an alternative therapeutic strategy for basal-like breast cancer patients.

## Introduction

Breast cancer is a heterogeneous disease, with discrete subtypes that can—in part—be characterized by their pathogenesis, molecular markers, and phenotype [1]. Five major breast cancer subtypes have been identified through mRNA profiling efforts: luminal A, luminal B, HER2-positive, basal-like, and claudin-low [2], [3]. Interestingly, the claudin-low subtype displays molecular features of EMT and cancer stem cells [4], [5], [6]. Consistently, the basal-like subtype, which largely overlaps with the triple-negative subtype, also exhibits aggressive clinical behavior and poor prognosis towards both targeted therapies and chemotherapy alike [7]. Compared to luminal, the basal-like subtype differentially expresses molecular markers such as epithelial cadherin (E-cadherin), vimentin, and fibronectin, commensurate with cells that have undergone EMT. This process gives cells a license to gain mobility and invade through the basement membrane that ultimately promotes the intravasation into the vasculature during the early stages of metastasis [8], [9]. There currently exists no approved targeted therapies against the triple-negative subtype of breast cancer, but the causal relationship between markers and clinical outcome of basal-like breast cancers suggests the potential for intervention [4], [5], [6]. Two microRNAs, miR-221 and miR-222, were previously identified by their highly discriminated expression patterns between the basal-like and the luminal subtypes of breast cancer [10]. More specifically, miR-221 and miR-222 levels are significantly increased in the basal-like subtype relative to the luminal subtype. miR-221/222 targeting of the transcription factor TRPS1 (tricho-rhino-phalangeal syndrome type 1) induced the EMT by releasing the negative regulation of ZEB2 [10]. However, as miRNAs have the potential to regulate multiple targets [11], we set out to find additional miR-221/222 targets that could regulate EMT in breast cancer cells.

Adiponectin receptor 1 (ADIPOR1) is one of several receptors for the adipocytokine adiponectin [12]. Adiponectin is made exclusively by adipocytes, and affects insulin sensitivity and proliferation in a variety of cell types [13]. Low levels of adiponectin are associated with obesity and higher risk of breast cancer [14], [15], while adiponectin has also been implicated with reducing breast cancer invasion via LKB1 signaling [16]. ADIPOR1 is a member of the progestin and adipoQ receptor (PAQR) family of cell-surface receptors which have seven transmembrane domains but do not couple to G-proteins. Instead, PAQRs are thought to be enzymes with intracellular, plasma membrane localized ceramidase activity. Recently, the ceramidase activity of ADIPOR1 has been shown to be required for protection of pancreatic β-cells and cardiomyocytes from fatty acid-induced apoptosis and for improved insulin sensitivity in obese mice through removal of hepatic ceramide [17]. The intracellular signaling pathways downstream of ADIPOR1, however, have not been well characterized. In this study, we uncovered a unique relationship between miR-221/222, ADIPOR1, and EMT and provide further insight into miR-221/222-mediated EMT and breast cancer pathogenesis.

## Materials and Methods

### Cell Lines, Tumors, and Reagents

All breast cell lines were procured from ATCC. The MDA-MB-231 cell line was maintained in RPMI containing 10% FBS supplemented with L-gluatamine, and the MCF10A cell line was maintained in Dulbecco’s Modified Eagle Medium/Ham’s F12 (DMEM/F12) media containing 5% horse serum, EGF (20 ng/ml), hydrocortisone (0.5 µg/ml), cholera toxin (100 ng/ml), and insulin (10 µg/ml). A supporting figure shows the short tandem repeat (STR) profiles for both cell lines used (Figure S2). Primary breast tumor samples were obtained from ILSbio, SeraCare Life Sciences, and Asterand. SMARTpool and individual siRNA, in addition to microRNA mimics, were purchased from Dharmacon. Cells were seeded at 50,000–100,000 cells/ml and transfected with RNAiMAX or Lipofectamine 2000 (Invitrogen). siRNA was transfected at a concentration of 100 nM, unless otherwise indicated. Transfection efficiencies of ≥75% were achieved as assessed by Green Fluorescent Protein (GFP) control. Site-directed mutagenesis (Stratagene) was performed according to the manufacturer’s protocol, and luciferase activity was measured by Dual-Glo reagents (Promega). Cucurbitacin (Sigma-Aldrich) and AG490 (Axon Medchem) were used at a concentration of 1 µM, and all neutralizing antibodies (R&D Systems) were used at a concentration of 20 µg/ml. ADIPOR1 overexpression, tagged cDNA clone (Origene) was stably selected at 500 µg/ml Geneticin (Invitrogen).

### Microarray and Quantitative Real Time PCR

RNA was purified using RNeasy (Qiagen) or Cells-to-CT (Ambion). For mRNA microarray, RNA was run on Affymetrix HGU133P2 chips, and mRNA from 55 basal and luminal breast cancer cell lines were profiled. For qPCR, cDNA was generated with the High Capacity RNA-to-cDNA Kit (Roche). cDNA was diluted at 1∶100, and used for 20ul total reactions on the 7900HT Fast Real-Time PCR System (Applied Biosystems). Primers from TaqMan Gene Expression Arrays (Applied Biosystems) were used: GAPDH (Hs02758991_g1), ADIPOR1 (Hs01114951_m1), E-cadherin (Hs01023895_m1), Vimentin (Hs00185584_m1), and Fibronectin (Hs00415006_m1). Reactions were performed in duplicate, and delta-delta-Cycle Threshold (ddCt) values were calculated after normalization to GAPDH. All microarray gene expression data for the breast cancer cell lines can be obtained from Gene Expression Omnibus (www.ncbi.nlm.nih.gov/geo) with accession number GSE12790.

### Western Blot Analysis and Immunofluorescence

For Western Blot Analysis, all cells were lysed with RIPA buffer supplemented with cOmplete, Mini, EDTA-free Protease Inhibitor Cocktail Tablets (Roche) and Halt Protease Inhibitor Cocktail (Thermo Scientific) and subsequently clarified by centrifugation. Protein concentration was quantified with the BCA Protein Assay Kit (Pierce). Reduced lysates were resolved on NuPAGE 4–12% Bis-Tris Gels (Invitrogen) and transferred with the Trans-Blot Turbo Transfer System (Bio-Rad). Membranes were blocked and incubated in 5% bovine serum albumin (BSA). The following antibodies were used: E-cadherin (BD Bioscience), Vimentin (Santa Cruz), Actin (Cell Signaling), p-IKKα/β Ser176/180 (Cell Signaling), IKKα (Cell Signaling), IKKβ (Cell Signaling), p-IκBα Ser32 (Cell Signaling), IκBα (Cell Signaling), p-STAT3 Tyr705 (Cell Signaling), STAT3 (Cell Signaling), and anti-Flag M2 (Sigma-Aldrich). For immunofluorescence, cells were transfected in an eight chamber glass slide, fixed in ice cold methanol for 5 minutes at –20°C and permeabilized in acetone for 2 minutes at –20°C. Cells were then stained with anti-Vimentin (Sigma-Aldrich) at room temperature for 1 hour followed by anti-mouse-cy3 (Jackson Lab) and Hoechst 33258. Cells were imaged and nuclei were quantitated on the Cellomics VTI Arrayscan.

### Invasion and Cell Growth Assays

For MCF10A invasion assays, 5×10^4^ cells were seeded on the upper side of 24-well BD BioCoat Matrigel Invasion Chamber inserts (BD Biosciences) 3 days post-transfection, and allowed to invade towards 10% horse serum. After 24 hours, cells on the upper side of the membrane were mechanically removed. Cells that invaded to the lower side were fixed and stained using the Diff-Quik Kit (Siemens). The membranes were photographed and the cells counted. For MDA-MB-231 cells, the high-throughput scratch CellPlayer 96-Well Kinetic Cell Invasion Assay (Essen Bioscience) was used according to the manufacturer’s protocol. 5×10^4^ cells were seeded, and the upper-layer of Matrigel was diluted to a concentration of 6 mg/ml. Images were collected via IncuCyte every 3 hours.

To assay growth rates, cells were first seeded into 96-well plates, with each well containing 2×10^3^ cells. The cells were then imaged every 3 hours for confluency percentage on an IncuCyte to obtain growth curves.

### Statistical Analysis

Spearman rank correlation coefficients or Student’s *t-test* calculations were used to determined statistical significance using GraphPad Prism.

## Results

### ADIPOR1 is a direct target of miR-221/222 and negatively regulates EMT

**Figure 1:**
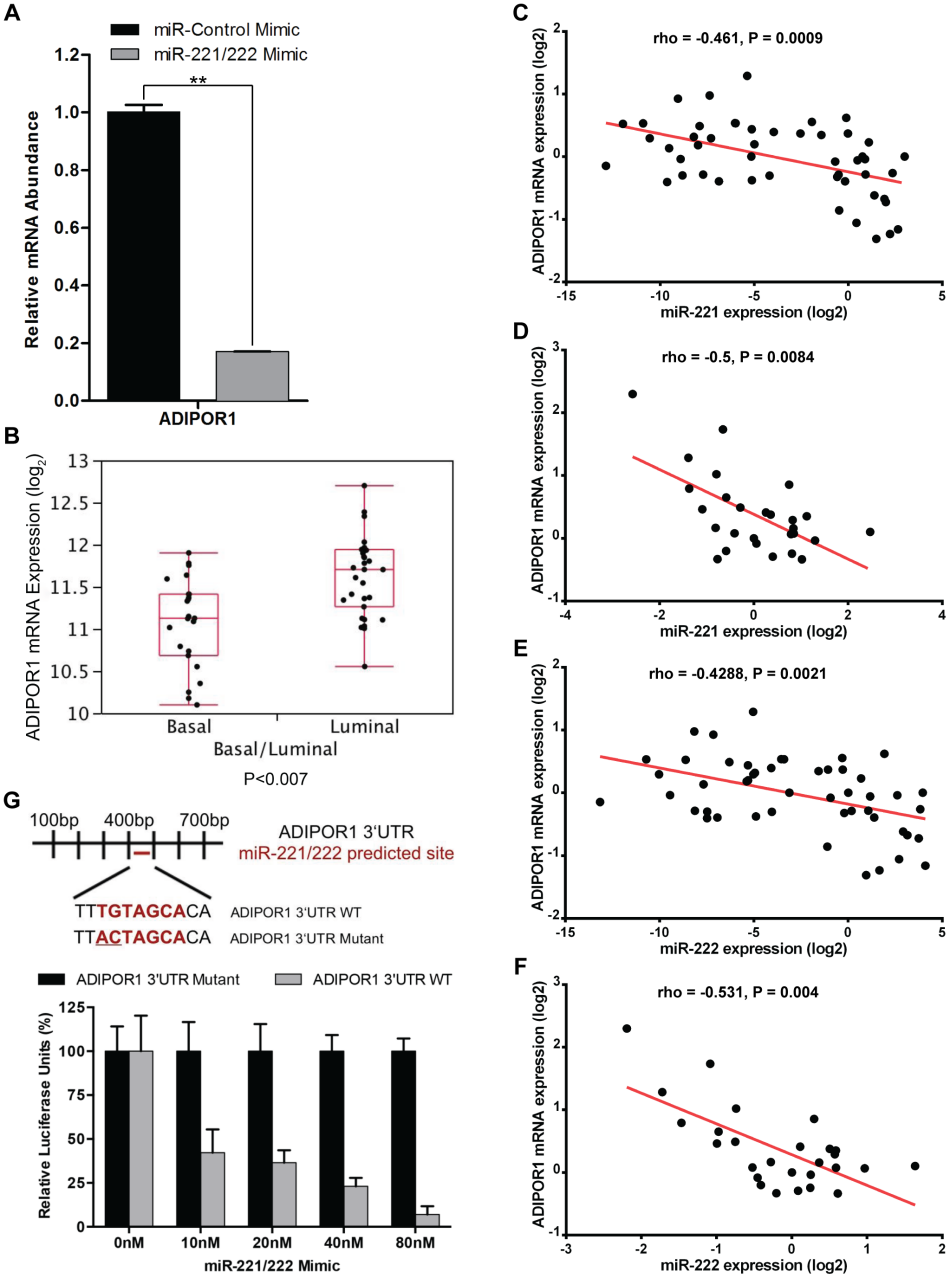
ADIPOR1 is a direct target of miR-221/222. (A) qRT-PCR analysis of MCF10A cells, 48 hours post transfection with either miR-Control or miR-221/222 mimic. Data represent means of triplicates ± SD. (B) ADIPOR1 mRNA expression data taken from microarray analysis of 32 luminal and 23 basal-like breast cancer cell lines. Data represented as boxplot, with whiskers indicating maximum and minimum values of the data set. (C) qRT-PCR analysis of ADIPOR1 mRNA expression versus miR-221 levels in 49 breast cancer cell lines. (D) qRT-PCR analysis of ADIPOR1 mRNA expression versus miR-221 levels in 27 primary breast tumors. (E) qRT-PCR analysis of ADIPOR1 mRNA expression versus miR-222 levels in 49 breast cancer cell lines. (F) qRT-PCR analysis of ADIPOR1 mRNA expression versus miR-222 levels in 27 primary breast tumors. (G) The predicted ADIPOR1 wild-type (WT) and mutant 3’UTR sequence for miR-221/222 is shown above. Below, MCF10A cells were co-transfected with the reporter constructs and an increasing titration of miR-221/222 mimic. Data represent means of triplicates ± SD.

Because microRNAs have the potential to target multiple genes when eliciting a phenotype, we wished to determine if other genes might be involved in promoting miR-221/222-mediated EMT. ADIPOR1 was predicted to be a target for miR-221/222 based on sequence prediction algorithms [18]. We transfected an immortalized, non-transformed human mammary epithelial cell line, MCF10A, with synthetic oligo mimics for miR-221/222 or with a scrambled miR mimic to function as a negative control (miR-Control), and observed that miR-221/222 treatment decreased ADIPOR1 mRNA levels by quantitative real time polymerase chain reaction (qRT-PCR) (Figure 1A). In addition, ADIPOR1 mRNA abundance was higher in luminal cell lines compared to basal-like cell lines(Figure 1B). Furthermore, miR-221 and miR-222 expression was negatively correlated with ADIPOR1 mRNA levels across breast cancer cell lines as well as tumors derived from breast cancer patients (Figures 1C – F), suggesting that ADIPOR1 may be an endogenous target of miR-221/222. However, to determine if ADIPOR1 is indeed a direct target of miR-221/222, we performed site-directed mutagenesis on nucleotides 1 and 2 of the predicted seed sequence within the conserved 3’UTR of ADIPOR1 (Figure 1C). The wild-type and mutant 3’UTR of ADIPOR1 were then cloned into a luciferase reporter construct [10]. Relative to the scrambled miR mimic, miR-221/222 decreased luciferase expression of the wild-type construct in a dose-dependent manner, but was unable to perturb the luciferase expression of the mutant 3’UTR construct in MCF10A cells (Figure 1C).

**Figure 2:**
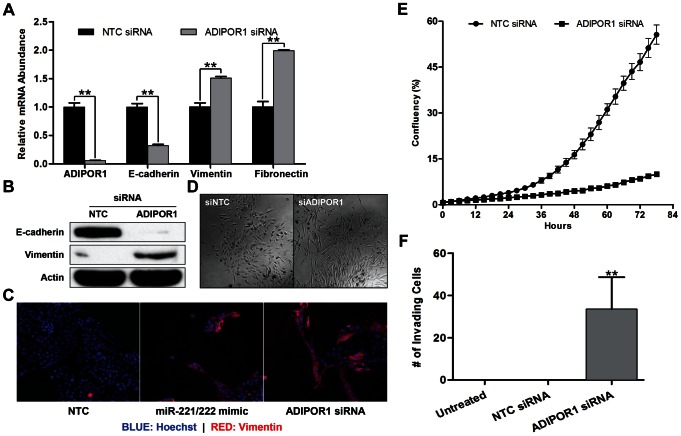
ADIPOR1 negatively regulates the EMT. (A) qRT-PCR analysis of MCF10A cells, 48 hours post transfection with either non-targeting control (NTC) or ADIPOR1 siRNA. Data represent means of triplicates ± SD; ** = P<0.001. (B) Western Blot analysis of E-cadherin and vimentin 5 days post siRNA transfection in MCF10A cells. (C) Immunofluorescence of vimentin and Hoechst staining in MCF10A cells 5 days post mimic or siRNA transfection. (D) Phase-contrast images of MCF10A cells 5 days post siRNA transfection. (E) Growth curve of MCF10A cells. Cells were initially transfected in a 6-well plate for 3 days prior to being transferred into a 96-well plate to assess growth rates. Data represent means of octuplets ± SD. (F) Invasion of MCF10A cells was assessed by a Boyden chamber invasion assay 3 days post siRNA transfection. Data represent means of quadruplicates ± SD; ** = P<0.001.

Since miR-221/222 has previously been shown to promote EMT [10], we wished to determine if ADIPOR1 silencing by small interfering ribonucleic acid (siRNA) would have a similar effect on molecular markers of EMT by qRT-PCR and western blotting. Dharmacon siRNA #2 produced the strongest knockdown of ADIPOR1 by mRNA, and this correlated with the largest decrease in E-cadherin and increase in vimentin relative to the other siRNAs in the pool; similar effects were seen with other siRNAs tested (Figure S1). All subsequent ADIPOR1 siRNA assays were therefore performed with Dharmacon siRNA #2. Indeed, ADIPOR1 siRNA downregulated E-cadherin and upregulated vimentin and fibronectin mRNA (Figure 2A). To confirm that mRNA levels correlated with protein expression, we performed a western blot with antibodies to E-cadherin and vimentin against samples treated with either non-targeting control (NTC) or ADIPOR1 siRNA and observed reduction in E-cadherin and elevation in vimentin protein expression (Figure 2B). Vimentin protein expression was also increased by both miR-221/222 mimic and ADIPOR1 siRNA treatment as determined by immunofluorescence (Figure 2C). Moreover, MCF10A cells transfected with ADIPOR1 siRNA adopted a more elongated and fibroblastic cellular morphology, consistent with a mesenchymal phenotype (Figure 2D). We also sought to determine whether cell invasion was enhanced by knocking down ADIPOR1. Despite attenuated growth rates, an observation that is consistent with EMT, we observed an increase in the invasion of MCF10A cells transfected with ADIPOR1 compared to NTC siRNA towards 10% serum (Figures 2E and 2F).

**Figure 3:**
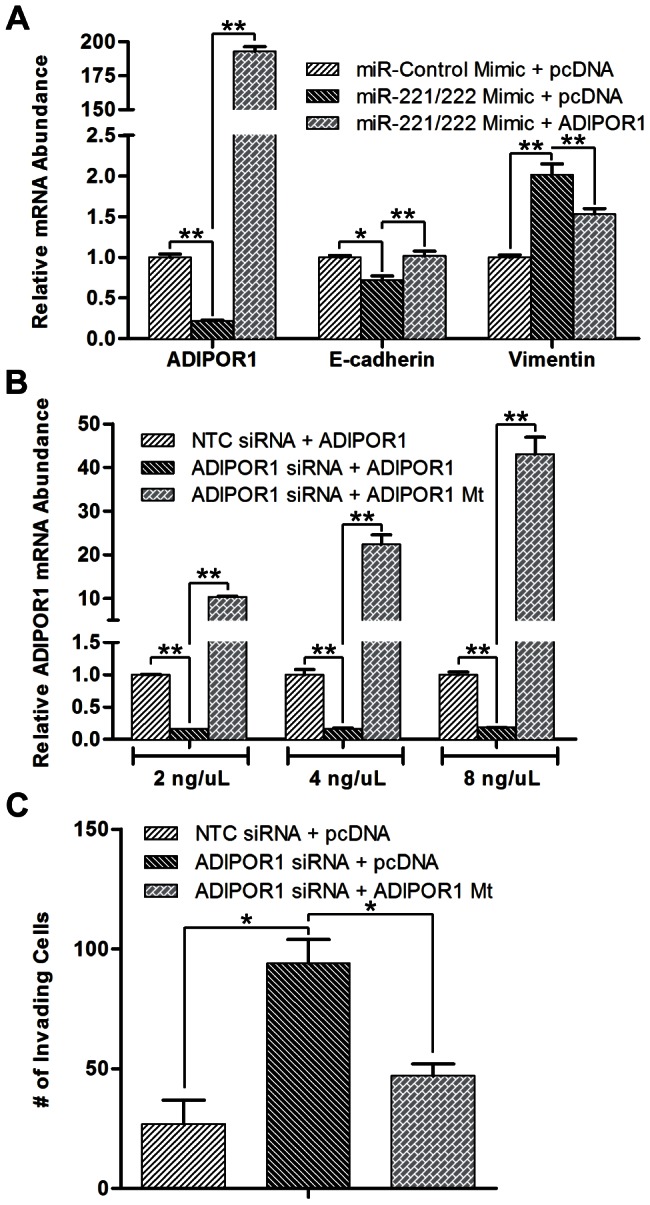
ADIPOR1 wildtype and siRNA mutant plasmids rescue miR-221/222 mimic and ADIPOR1 siRNA-induced EMT, respectively. (A) qRT-PCR analysis of MCF10A cells, 48 hours post co-transfection with plasmid (1 µg/ml) and mimic. Data represent means of triplicates ± SD; * = P<0.05, ** = P<0.001. (B) qRT-PCR analysis of ADIPOR1 mRNA in MCF10A cells, 48 hours post co-transfection of siRNA and titrated mutant plasmid. Data represent means of triplicates ± SD; ** = P<0.001. (C) Invasion of MCF10A cells as assessed by a Boyden chamber invasion assay 3 days post siRNA and plasmid (100 ng/ml) co-transfection. Data represent means of quadruplicates ± SD; * = P<0.05.

To confirm that ADIPOR1 was not only a target of miR-221/222, but also rescues a miR-221/222 mimic-induced EMT in MCF10A cells, we co-transfected an ADIPOR1 overexpression plasmid lacking its native 3’UTR with miR-221/222 mimic and indeed saw that increased ADIPOR1 levels rescued E-cadherin and vimentin expression (Figure 3A). To rule out the possibility ADIPOR1 siRNA has substantial off-target effects, we generated a siRNA resistant mutant of ADIPOR1 and found that not only was it able to rescue ADIPOR1 expression in a dose-dependent manner, but also blunted the number of invading MCF10A cells caused by transfection of ADIPOR1 siRNA (Figure 3B and 3C).

Collectively, these data suggest that ADIPOR1 is a target of miR-221/222, ADIPOR1 abundance negatively correlates with EMT markers and phenotypes, and depletion of ADIPOR1 can elicit molecular and phenotypic properties associated with EMT.

### ADIPOR1 protects against the EMT by negatively regulating the NF-κB, IL6, and JAK2/STAT3 signaling axis

**Figure 4:**
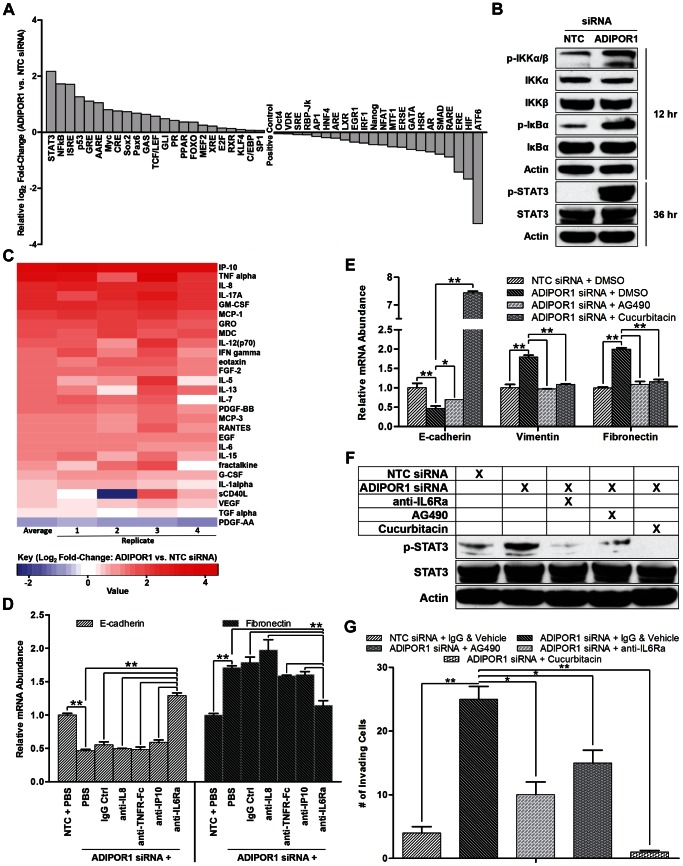
ADIPOR1 protects against the EMT by negatively regulating the NF-κB, IL6, and JAK2/STAT3 signaling axis. (A) Cignal 45-Pathway Reporter Array of MCF10A cells 3 days post siRNA transfection. (B) Western Blot analysis of canonical NF-κB markers and STAT3 in MCF10A cells. Hours on right indicate time post siRNA transfection. (C) Heatmap representation of Millipore Luminex cytokine array, with conditioned media from MCF10A cells collected 5 days post siRNA transfection and normalized to cell number. (D) qRT-PCR analysis of MCF10A cells, 48 hours post co-treatment of neutralizing antibody and siRNA. Data represent means of triplicates ± SD; ** = P<0.001. (E) qRT-PCR analysis of MCF10A cells, 48 hours post co-treatment of drug and siRNA. Data represent means of triplicates ± SD; * = P<0.05, ** = P<0.001. (F) Western Blot analysis of phosphorylated and total STAT3 in MCF10A cells upon combination drug/neutralizing antibody and siRNA treatment. Lysate collected 36 hours post treatment. (G) Invasion of MCF10A cells as assessed by a Boyden chamber invasion assay 3 days post combination drug/neutralizing antibody and siRNA treatment. Data represent means of quadruplicates ± SD; * = P<0.05, ** = P<0.001..

Next we sought to determine a mechanism of action for ADIPOR1 suppression of the EMT. Transfecting ADIPOR1 or NTC siRNA into MCF10A cells in 96-well Cignal 45-Pathway Reporter Arrays revealed a number of upregulated and downregulated pathways (Figure 4A). Notably, the two highest expressed reporters were STAT3 and NF-κB, both well-known drivers of the EMT in breast cancer, as well as other cancers [19], [20], [21], [22]. To confirm the results from the Cignal 45 reporter assay, we assessed markers of canonical NF-κB and STAT3 signaling by western blot. I KappaB Kinase α/β (IKKα/β) and Inhibitor of κB α (IκBα) phosphorylation increased 12 hours post ADIPOR1 siRNA transfection, and STAT3 phosphorylation increased after 36 hours (Figure 4B). The temporal phosphorylation of canonical NF-κB and STAT3 suggested that there might exist secondary messengers to allow for crosstalk and regulation between the two signaling nodes. To address that question, we performed a 41-plex Luminex secreted factor array on conditioned media after ADIPOR1 targeting by siRNA. Conditioned media was collected 5 days post transfection and 28 cytokines and growth factors were detectable within the linear range of the assay (Figure 4C). Noteworthy was the increased abundance of all but one analyte in response to ADIPOR1 knockdown, reminiscent of a quasi-cytokine storm or hypercytokinemia, and consistent with classic canonical NF-κB activation [23]. Co-treatment of neutralizing antibodies against the top three upregulated cytokines in addition to IL6—a well-documented mediator of EMT via Janus Activated Kinase (JAK)/STAT3 signaling [24], [25], [26]—with siRNA targeting ADIPOR1 revealed the unexpected result that only anti-IL6Ra was able to blunt an ADIPOR1 siRNA-induced EMT. This suggested that IL6 is a required, intermediate secondary messenger produced after NF-κB activation, which subsequently induces STAT3 phosphorylation in an autocrine manner (Figure 4D). Furthermore, small-molecule inhibitors against JAK2 (AG490) and STAT3 (Cucurbitacin) were able to attenuate an ADIPOR1 siRNA-mediated EMT (Figure 4E) [27], [28], [29]. Because STAT3 has been implicated as a key driver for the EMT, we tested whether STAT3 phosphorylation could be perturbed with the neutralizing antibody anti-IL6Ra, and the small-molecule inhibitors AG490 and Cucurbitacin. We observed attenuated phospho-STAT3 levels when the inhibitors and neutralizing antibody were treated in concert with siRNA targeting ADIPOR1 in MCF10A cells (Figure 4F). Intriguingly, we also noted a reduction in cell invasion, with correlation to relative STAT3 phosphorylation (Figures 4F and 4G). Put together, we propose that ADIPOR1 protects against the EMT by suppressing the NF-κB, IL6, and JAK2/STAT3 signaling axis.

### ADIPOR1 overexpression attenuates the EMT and invasion

**Figure 5:**
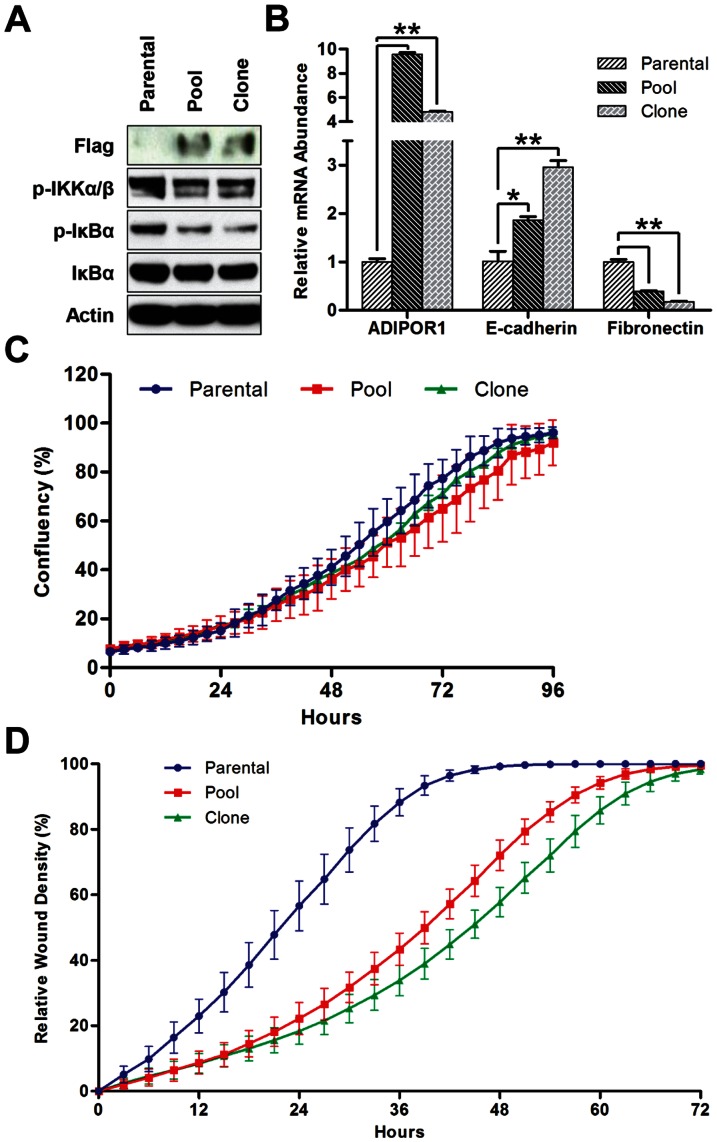
ADIPOR1 overexpression attenuates the EMT and invasion. (A) Western Blot analysis of anti-Flag and canonical NF-κB markers in MDA-MB-231 cells 2 days after passaging cells. (B) qRT-PCR analysis of MDA-MB-231 cells 2 days after passaging cells. Data represent means of triplicates ± SD; * = P<0.05, ** = P<0.001. (C) Growth curve of MDA-MB-231 cells. Data represent means of octuplets ± SD. (D) Invasion of MDA-MB-231 cells as assessed by the CellPlayer 96-Well Kinetic Cell Invasion Assay. Data represent means of octuplets ± SD.

To further confirm ADIPOR1 as a regulator of the EMT in breast cancer cell lines, we sought to overexpress the receptor. We stably transfected an ADIPOR1, Myc-DDK-tagged, construct into the basal-like breast cancer cell line MDA-MB-231, and generated a pool of polyclonal stable lines, in addition to a single clone isolated from the pool. Consistent with previous results, we saw decreased phosphorylation of both canonical markers of NF-κB, IKKα/β and IκBα, in the pool and clone compared to the parental MDA-MB-231 cells (Figure 5A). We also detected an increase in E-cadherin and a decrease in fibronectin abundance, suggesting that ADIPOR1 overexpression promotes the mesenchymal-to-epithelial transition (MET) (Figure 5B). Finally, we wished to investigate how ADIPOR1 expression would affect cell invasion. Despite similar growth curves, the pool and clone derivatives reduced invasion compared to parental MDA-MB-231 cells (Figures 5C and 5D). In addition, cell invasion rates correlated well with the degree of MET induction. Together, these data suggest that ADIPOR1 abundance in the luminal-subtype of breast cancers protects against the EMT, and this protection can be conferred by overexpression in the basal-like subtype.

## Discussion

Breast cancer is the second most common cancer worldwide after lung and the leading cause of cancer deaths in women [30]. The triple negative subtype of breast cancer, with its poor prognosis and lack of approved targeted therapies, remains a significant unmet medical need. Recently, small RNA molecules known as miRNAs have emerged as post-transcriptional regulators of mRNA stability, and have been identified to enhance several aspects of breast cancer pathogenesis including metastasis [31], invasion [32], and self-renewal [33]. Each of these phenotypes can be further modulated by the EMT program [34]. miR-221/222 has previously been reported to promote the EMT by negatively regulating TRPS1 that leads to increased ZEB2 expression [10]. Here, we found that miR-221/222 additionally targets ADIPOR1 and abrogates ADIPOR1 inhibition of the EMT. Our data show that miR-221/222 simultaneously targets two genes, ADIPOR1 and TRPS1, which likely allows for tight control of the EMT. Both the NF-κB/IL6/JAK2/STAT3 cascade, which we show here is modulated by miR-221/222-mediated downregulation of ADIPOR1, and ZEB2 expression, which we have previously shown is repressed by TRPS1, are well-characterized effector pathways in cancer metastasis, invasion, and EMT. It is intriguing that altering TRPS1 or ADIPOR1 alone is sufficient to modulate an EMT phenotype since there is very little evidence that the two pathways are directly related; however, there are many ways to induce EMT and the fact that miR-221/222 is targeting two independent pathways that, together, are signaling to nodes key for mediating EMT in breast cancer, underscores the importance of our findings and warrants further investigation. One potential limitation of our study is that most of our work was done in two cell lines, MCF10A and MDA-MB-231, and could limit the generalization of our findings; however, the negative correlation between miR-221 and miR-222 expression and ADIPOR1 mRNA expression in breast tumors as well as in breast cancer cell lines suggests that our model could hold true in other systems.

Genetic variants of ADIPOR1 and its ligand adiponectin have been associated with increased risk of breast cancer [35] and circulating adiponectin levels are inversely correlated with breast cancer in post-menopausal women [15], while higher levels of adiponectin are associated with improved survival in obese, insulin-resistant breast cancer patients [36]. In mouse models of mammary gland tumors, adiponectin has been proposed to play an indirect role in tumor growth, through the regulation of non-tumor endothelial cells and angiogenesis [37]. This activity may be linked to the observed overexpression of T-cadherin, the most well-defined adiponectin receptor [38], in endothelial vascular cells of breast tumors [39], [40]. Our data do not directly implicate adiponectin in the regulation of the EMT in the luminal-subtype of breast cancer cell lines. In fact, we suspect that, as ADIPOR1 is an enzyme with intrinsic activity, the loss of this activity in cells is sufficient to activate the EMT program in an adiponectin-independent manner. None of the experiments described here use exogenous, recombinant adiponectin, although the presence of adiponectin in serum means we cannot rule out some role for and contributions from adiponectin in the activities described.

Consistent with previous reports, we have demonstrated NF-κB and IL6 as drivers of the EMT phenotype. The canonical NF-κB pathway is required for induction and maintenance of EMT in a murine mammary carcinoma model; specifically, it has been shown that activation of NF-κB signaling promoted EMT in the absence of TGF-beta, whereas inhibition of NF-κB signaling prevented and reversed EMT [22]. Recombinant IL6 treatment or stable IL6 overexpression generated an EMT via the JAK/STAT3/Snail signaling pathway, and IL6 overexpression displayed increased metastasis in a severe combined immunodeficiency (SCID) mouse xenograft model [26]. In the epithelial-like T47D breast cancer cells, IL6-induced EMT generated CD44+ cells with stem-like properties [25]. Not surprisingly, IL6 is highly expressed in claudin-low tumors and is part of the claudin-low signature [6]. Along with its well-documented role in controlling the initiation and progression of human cancers [41], [42], NF-κB signaling has also been implicated in drug resistance against both targeted therapies and chemotherapy alike [43], [44], [45]. Tumor cells of breast cancer patients that survive after conventional chemotherapy treatment often exhibit molecular markers consistent with a mesenchymal phenotype [4]; meanwhile, exposure of luminal-subtype breast cancer cells to chemotherapy results in shifts of traits towards that of basal-like cells [46]. In our previous study on miR-221/222, treatment of basal-like breast cancer cells with a mitogen-activated protein/extracellular signal-regulated kinase kinase (MEK) inhibitor resulted in MET and alludes to the strategy that dual treatment with MEK and NF-κB or JAK2 inhibitors may be able to dampen metastasis and sensitize previously resistant and refractory cells to chemotherapy [10]and suggest that patients with basal-like breast cancer may benefit with a MEK inhibitor combined with a/NF-κB or JAK2 inhibitor.

Collectively our data may serve to help unravel the complexities behind EMT signaling and the miR-221/222-mediated EMT mechanism of action. Further studies towards identifying the signaling intermediates of, and binding partners for, ADIPOR1 could additionally provide an avenue for therapeutic intervention by revealing novel targets for drug design for the treatment of basal-like breast cancers.

## Supporting Information

Figure S1 **Deconvolution of Dharmacon and Qiagen ADIPOR1 siRNA in MCF10A cell lines.** siRNA oligos were transfected into MCF10A cells for 48 hours and ADIPOR1 mRNA levels were determined by qRT-PCR analysis.
(PDF)

Figure S2 **STR profiles for the MCF10A and MDA-MB-231 cell lines used in this study.**
(PDF)
