# Supplementary figures and images for: miR-221/222 Targets Adiponectin Receptor 1 to Promote the Epithelial-to-Mesenchymal Transition in Breast Cancer
Source: PLoS One. 2013 Jun 11;8(6):e66502. doi: 10.1371/journal.pone.0066502 (PMC3679042; doi:10.1371/journal.pone.0066502)

## Dharmacon ADIPOR1 siRNAs

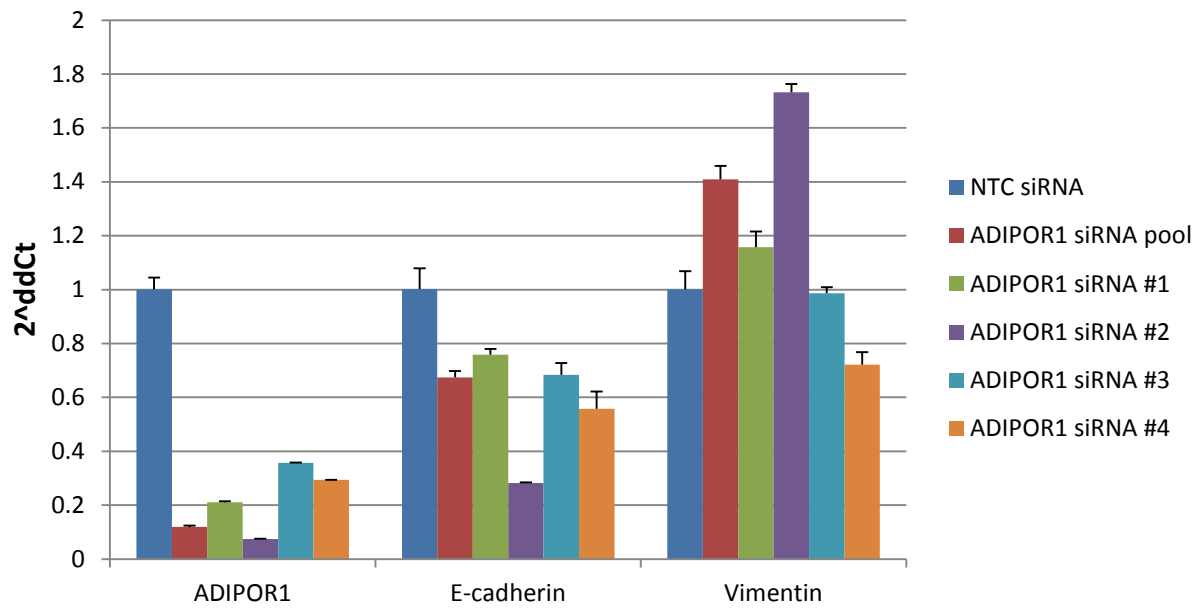

## Qiagen ADIPOR1 siRNAs

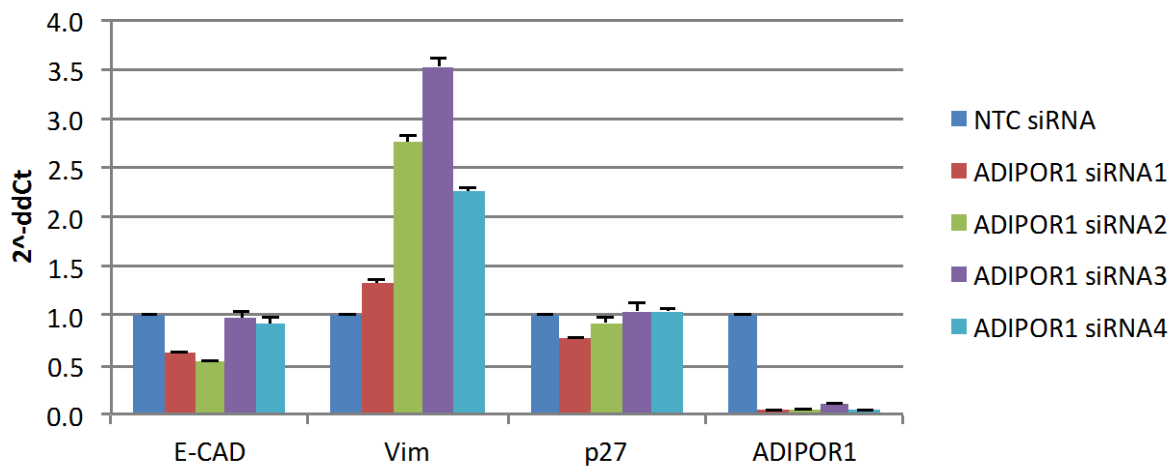

Supplement: Figure S1 — Deconvolution of Dharmacon and Qiagen ADIPOR1 siRNA in MCF10A cell lines. siRNA oligos were transfected into MCF10A cells for 48 hours and ADIPOR1 mRNA levels were determined by qRT-PCR analysis. (PDF) [file pone.0066502.s001.pdf]
