# Supplementary material for: miR-221/222 Targets Adiponectin Receptor 1 to Promote the Epithelial-to-Mesenchymal Transition in Breast Cancer
Source: PLoS One. 2013 Jun 11;8(6):e66502. doi: 10.1371/journal.pone.0066502 (PMC3679042; doi:10.1371/journal.pone.0066502)
Supplement: Figure S2 — STR profiles for the MCF10A and MDA-MB-231 cell lines used in this study. (PDF) [file pone.0066502.s002.pdf]

STR Profiles for Cell Lines Used

CLID	Cell Line Name	cName	D3S1358	TH01	D21S11	D18S51	Penta E	D5S818	D13S317	D7S820	D16S539	CSF1PO	Penta D	AMEL	vWA	D8S1179	TPOX	FGA
131434	MCF 10A	MCF 10A	14, 18	8, 9.3	28, 30	18, 19	13, 14	10, 13	8, 9	10, 11	11, 12	10, 12	10, 12	X	15, 17	14, 16	9, 11	22, 24
583925	MDA-MB-231	MDA-MB-231	16	7, 9.3	33.2	11, 16	11	12	13	8, 9	12	12, 13	11, 14	X	15, 18	13	8, 9	22, 23
